# Evidence-Based Brief Psychological Treatment for Emotional Disorders in Primary and Specialized Care: Study Protocol of a Randomized Controlled Trial

**DOI:** 10.3389/fpsyg.2018.02674

**Published:** 2019-01-08

**Authors:** Mario Gálvez-Lara, Jorge Corpas, José Fernando Venceslá, Juan A. Moriana

**Affiliations:** ^1^Department of Psychology, University of Córdoba, Córdoba, Spain; ^2^Maimónides Biomedical Research Institute of Córdoba, Córdoba, Spain; ^3^Reina Sofia University Hospital, Córdoba, Spain

**Keywords:** emotional disorders, brief psychological treatment, transdiagnostic, primary care, randomized controlled trial

## Abstract

Emotional Disorders (EDs) are very prevalent in Primary Care (PC). However, general practitioners (GPs) have difficulties to make the diagnosis and the treatment of this disorders that are usually treated with drugs. Brief psychological therapies may be a new option to treat EDs in a PC context. This article aims to present a study protocol to evaluate the effectiveness and the efficiency of an adaptation to brief format of the “Unified Protocol (UP) for the transdiagnostic treatment of EDs.” This is a single-blinded RCT among 165 patients with EDs. Patients will be randomly assigned to receive brief psychological treatment based on UP, conventional psychological treatment, conventional psychological treatment plus pharmacological treatment, minimum intervention based on basic psychoeducational information, or pharmacological treatment only. Outcome measure will be the following: GAD-7, STAI, PHQ-9, BDI-II, PHQ-15, PHQ-PD, and BSI-18. Assessments will be carried out by blinded raters at baseline, after the treatment and 6-month follow-up. The findings of this RCT may encourage the implementation of brief therapies in the PC context, what would lead to the decongestion of the public health system, the treatment of a greater number of people with EDs in a shorter time, the reduction of the side effects of pharmacological treatment and a possible economic savings for public purse.

**Clinical Trial Registration:** ClinicalTrial.gov, identifier NCT03286881. Registered September 19, 2017.

## Introduction

### Background

Nowadays, concern for mental health disorders and problems has increased significantly worldwide (Mental Health Foundation, 2016). Common mental health disorders, also called emotional disorders (EDs), are growing exponentially in recent decades (Vos et al., 2015; Chisholm et al., 2016). These disorders include DSM-5 (American Psychiatric Association, 2013) diagnostics of depression, generalized anxiety disorder, panic disorder, obsessive-compulsive disorder, post-traumatic stress disorder, social anxiety disorder, and specific phobias (National Institute for Health, and Clinical Excellence [NICE], 2011). EDs are very prevalent in primary care (PC) services (Roca et al., 2009; Lejtzén et al., 2014), since about 40% PC consultations would be direct or indirect related with this kind of problems (Kroenke et al., 2007).

Some researches point out the difficulties presented by general practitioners (GPs) to make the diagnosis and the treatment of EDs (Collings, 2005; Aragonés et al., 2006; Fernández et al., 2006; DeVicente and Berdullas, 2009; Gerrits et al., 2013; Jacka et al., 2013), and warn of the excess in the use of drugs (Secades Villa et al., 2003). Patients with mild-moderate symptoms of EDs are usually treated in PC with drugs as the first therapeutic option (Kovess-Masfety et al., 2007), which contradicts the research and the international clinical guides instructions (National Institute for Health, and Clinical Excellence [NICE], 2011), that suggest psychological therapies as treatment of choice. Although pharmacological treatment is obviously of choice in severe cases, scientific research indicates that many of the patients with mild-moderate symptoms could reduce them with psychological treatments, without medication (Moreno and Moriana, 2012) and that psychological treatment is more effective than medication in these patients in a PC context (Watts et al., 2015). In the same way, it is suggested that, independently of the severity of the case, psychological treatment should always be present (Gonçalves et al., 2003; National Institute for Health, and Clinical Excellence [NICE], 2011). Other studies have concluded that psychological treatment applied in PC for this kind of patients promotes a greater recovery, improves the quality of care, decreases the prescription of drugs and reduces the hyperfrequency (Wells et al., 2000; Patel et al., 2010). Furthermore, psychological treatment is mostly preferred by patients, since it is in accordance with their conception of the problems they suffer (Prins et al., 2008; Walters et al., 2008).

Despite the importance of evaluating the efficacy of psychological treatments (Moriana et al., 2017; Gálvez-Lara et al., 2018), the knowledge of the empirical research’s results and its later integration by the professional in the clinical practice does not get to consolidate completely in the applied fields (Herbert, 2003; Kazdin, 2008, 2011; Barlow et al., 2013). In this line, some studies have evidenced that psychologist, often, do not use evidence-based treatment (Lilienfeld, 2010; Dobson and Beshai, 2013; Dozois, 2013). At other times, psychologists do not use all the key elements of evidence-based treatment (Stobie et al., 2007). For these reasons, some authors have manifested the existence of a gap between the applied psychology and the scientific research (Westen et al., 2004; Kazdin, 2008, 2011; Babione, 2010).

Some surveys have indicated that applied psychologists feel that research’s findings do not show the reality of clinical practice (Tasca et al., 2015) and affirm that the manualized treatments are very rigid for the habitual clinical practice (Gyani et al., 2015). This situation has promoted the demand for a better adequacy of the treatments to the real contexts of application (Kazdin, 2008), which entails their flexibility and use in different contexts (primary and specialized care) and their adaptation to brief formats of limited time (Moriana and Martínez, 2011).

### Brief Therapies: Adaptation of the Psychological Treatments to the Real Context of Application

In recent years, the possibility of adapting conventional psychological therapies to an abbreviated format as a possible solution for the correct treatment of EDs has been suggested (Shepardson et al., 2016). Brief therapy or “time limited therapy” emerges as a therapeutic option of low cost to respond to the demands of public health about the use of psychological therapies of short duration that generate favorable results in clinical practice (Hewitt and Gantiva, 2009). To be considered short, a therapy should have more than two sessions and less than ten, establishing an average of six sessions, on the basis of the idea that the guides are flexible to the characteristics and symptoms of the patient (Cape et al., 2010). Although there is no agreement among different authors about how many sessions include limited time therapies, all agree on the importance of the time as a therapeutic tool (Miller, 2000; MacNeil, 2001; Bedics et al., 2005; Hewitt and Gantiva, 2009; Lyons and Low, 2009). The limitation of the number of sessions helps both therapist and patient to focus fully on the therapy, increases the motivation of the patient and requires the establishment of achievable goals by the professional, considering each session as one intervention with a particular outcome with the aim that the patient undergoes change as soon as possible (Fosha, 2004).

Brief therapy responds adequately to the economic resources and psychological requirements of patients (Lyons and Low, 2009), and could be used to offer psychological therapy to patients who are in waiting lists for access to specialized programs, such as initial treatment for risk users and as a complement to more extensive psychological treatments (Sánchez and Gradolí, 2002). Due to its idiosyncrasy, brief therapies are especially indicated for adaptive and emotional problems of mild or moderate severity and it is suggested that they should be the first step for the therapeutic approach in this patient profile, giving a wide accessibility to patients and an effective response to their symptoms (Collings et al., 2015).

Some works have shown that brief therapies have obtained similar results to conventional therapies (Miller, 2000; Bloom, 2001; Lyons and Low, 2009; Nieuwsma et al., 2012), demonstrating its effectiveness in reducing anxiety and depressive symptoms (Koutra et al., 2010; Bernhardsdottir et al., 2013; Saravanan et al., 2017), on the improvement of problem-solving skills (Bannink, 2007), in reducing symptoms of posttraumatic stress disorder (Kip et al., 2016) or in the decrease in the intake of alcohol in people who had an excessive consumption (Gantiva et al., 2003). In addition, brief therapies have not only proved effective immediately after treatment, but also that the improvement in the patient stays long after the end of the intervention (Hamdan-Mansour et al., 2009; Vázquez et al., 2012).

### Unified Protocol for the Transdiagnostic Treatment of EDs

One of the main problems of the categorical approach to psychopathology is the high comorbidity among the different mental disorders (Sandín et al., 2012). In last years, a new approach has emerged for the treatment of mental disorders that aims to develop interventions that can be used to treat the common symptoms of various psychological disorders (Belloch, 2012). This approach, known as transdiagnostic, considers that mental disorders share numerous cognitive and behavioral processes that contribute to the development and maintenance of symptoms (Harvey et al., 2004). Thus, the transdiagnostic treatment is defined as a “therapy that is made available to individuals with a wide range of diagnosis and that does not rely on knowledge of these diagnoses to operate effectively” (Mansell et al., 2009, p. 14).

Psychological treatment from a transdiagnostic approach is especially appropriate for EDs, since accumulated finding have shown important shared characteristics among depression, anxiety, and other emotion-related disorders (Barlow, 2002; Barlow et al., 2004; Brown and Barlow, 2009; Rosellini et al., 2015). David Barlow and their team developed the “Unified Protocol for the transdiagnostic treatment of EDs” to address the underlying symptoms of disorders in which anxiety and emotional deregulation play an important role (Allen et al., 2008; Ellard et al., 2010; Barlow et al., 2011). The efficacy of PU has been widely demonstrated (Reinholt and Krogh, 2014), both in individual format (Farchione et al., 2012) as in group format (Bullis et al., 2015), with the possibility of simultaneous application to patients with a variety of disorders, so it could reduce the waiting lists and the cost of the individual treatment (Osma et al., 2018).

From a transdiagnostic perspective focused on brief therapies (less than ten session) for the treatment of the EDs, several studies have shown their effectiveness both for the reduction of symptoms and for the application of treatment to many patients with different characteristics and comorbid disorders (Roy-Byrne et al., 2010; Dear et al., 2011; Titov et al., 2011; Schmidt et al., 2012; Osma et al., 2015). However, these studies present some limitations related to their designs, since some of them lack a control group (single group design), and others use only a waitlist control group or medication (treatment as usual) as a comparator.

Due to the limitations of the previous studies, it is necessary to design a protocol that evaluates the efficacy of brief therapy from a transdiagnostic perspective compared to other treatment modalities that are frequently used in mental health services (e.g., conventional psychological treatment, combined treatment or psychoeducational information).

### The Present Study

The aim of this work is to examine the efficacy of several types of interventions, in therapeutic and cost-effectiveness terms, for the treatment of patients with EDs. The different interventions are the following: brief psychological treatment based on UP; conventional psychological treatment; conventional psychological treatment plus pharmacological treatment; minimum intervention based on basic psychoeducational information, counseling and bibliotherapy; and pharmacological treatment.

We hypothesize that “conventional psychological treatment plus pharmacological treatment” will be the most effective treatment, but “brief psychological treatment based on UP” will be the most efficient treatment (cost-effectiveness). Besides, we expect that psychological treatment (brief and conventional) will be more effective than pharmacological treatment, but less effective than “conventional psychological treatment plus pharmacological treatment,” and that “brief psychological treatment” will be as effective as “conventional psychological treatment” but more efficient than this one. Finally, we think that “minimum intervention based on basic psychoeducational information, counseling, and bibliotherapy” will be more effective than pharmacological treatment but will get worst results that different versions of psychological treatment.

## Materials and Equipment

### Study Design

**FIGURE 1:**
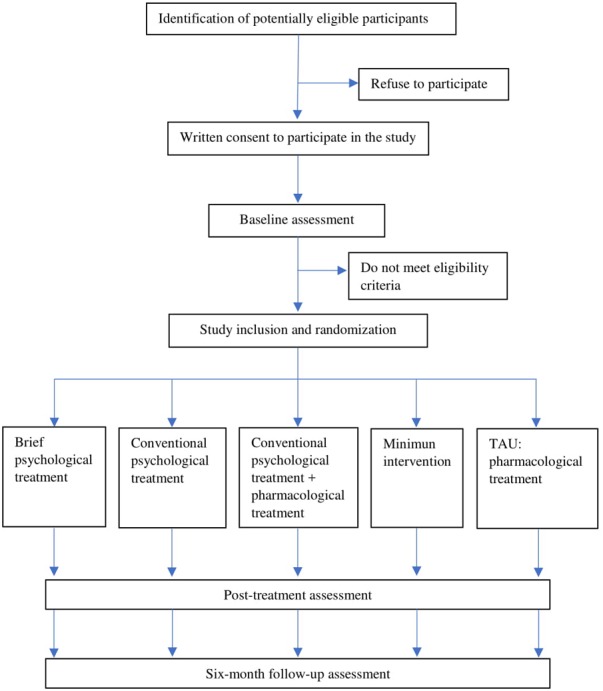
Flow chart of the overall study design.

This will be a multicenter randomized controlled trial (RCT) with five groups that will be realized in primary and specialized care centers from Córdoba (Spain) and its province. Patients will be randomly assigned to receive one of the following interventions: (a) *Brief psychological treatment based on UP*, (b) *Conventional psychological treatment*, (c) *Conventional psychological treatment plus pharmacological treatment*, (d) *Minimum intervention based on basic psychoeducational information, counseling and bibliotherapy*, and (e) *Active comparator treatment as usual (TAU): pharmacological treatment only*. For ethical reasons, in the case of low response to treatment, participants in the *Brief psychological treatment* and *Minimum intervention conditions* will be removed from the trial and will receive the treatment proposed by their GP. Outcome measures will be taken before randomization (baseline assessment), after the intervention (post-treatment assessment) and at 6-month follow-up. The overall study design is summarized in Figure 1. This RCT will be implemented following the SPIRIT guidelines (Chan et al., 2013a,b) and the CONSORT statement (Moher et al., 2010; Schulz et al., 2010).

### Instruments and Measures

#### GAD-7 Scale

The GAD-7 scale (Spitzer et al., 2006) is utilized to assess generalized anxiety disorder and other anxiety disorders. In this instrument, participants indicate the presence of anxiety symptoms during the last 2 weeks. The maximum score is 21. The scale has shown high internal consistency (α = 0.89–0.93) (García-Campayo et al., 2010; Zhong et al., 2015).

#### State-Trait Anxiety Inventory (STAI)

The STAI (Spielberger et al., 1983) is a frequently utilized instrument to assess state and trait anxiety. It is composed of 40 items, 20 to assess state anxiety and 20 to assess trait anxiety. A high score indicates the presence of severe anxiety symptoms. This scale has shown excellent internal consistency (α = 0.86–0.95) (Spielberger et al., 1983; Fonseca-Pedrero et al., 2012).

#### Patient Health Questionnaire-9 (PHQ-9)

The PHQ-9 (Kroenke et al., 2001) is a specific screening tool for depression in which participants respond to nine items on depressive symptoms during the last 2 week through a 4-point Likert scale. The intenal consistency of PHQ-9 ranged from 0.86 to 0.89 (Kroenke et al., 2001).

#### Beck Depression Inventory-Second Edition (BDI-II)

The BDI-II (Beck et al., 1996) is a commonly used scale to assess the presence of depressive symptoms in adolescents and adults. The BDI-II is composed of 21 items that cover the diverse symptons of major depressive disorder. Each item is scored on a scale from 0 to 3 (from lowest to highest severity), so the maximum score obtained can be 63 points. The intenal consistency shown by this inventoy has been excellent (α = 0.94) (Arnau et al., 2001).

#### Patient Health Questionnaire-15 (PHQ-15)

The PHQ-15 (Kroenke et al., 2002) is frequently used to measure the presence of somatic symptoms. The Spanish version is composed of 13 and the maximum score for this instrument can be 30 points. The PHQ-15 has shown an acceptable internal consistency (α = 0.78) (Ros Montalbán et al., 2010).

#### Patient Health Questionnaire-Panic Disorder (PHQ-PD)

The PHQ-PD will be use to measure panic disorder symptoms (Spitzer et al., 1999). It is composed of 15 items. We are facing a probable panic disorder when the patient answers affirmatively to the first four items and presents four or more symptoms related to this disorder.

#### Brief Symptom Inventory 18 (BSI-18)

The BSI-18 (Derogatis, 2000) is the shortest version of the Symptom-Checklist 90-R (Derogatis, 1994). It is composed of 18 items and contains three scales (anxiety, depression, and somatization) and a Global Severity Index (GSI). The internal consistency showed by this inventory has been good: GSI α = 0.93, anxiety α = 0.84, depression α = 0.87, and somatization α = 0.82 (Franke et al., 2017).

## Stepwise Procedures

### Sample Size

To establish the sample size, we considered the effect sizes shown by the previous literature. Due to this study pretends to examine the efficacy of several types of interventions, we collected data from previous findings about brief therapies, transdiagnostic treatment, UP, conventional CBT, and combined therapy for the treatment of anxiety disorders and depression. A meta-analytic study examined the effect of various brief psychological therapies in comparison to usual GP care (Cape et al., 2010). The results of the meta-analysis to brief cognitive behavior therapy showed an effect size (Cohen’s *d*) of 1.06 for anxiety, 0.33 for depression and 0.26 for mixed anxiety and depression. In the case of brief counseling, the meta-analysis showed and effect size (Cohen’s *d*) of 0.41 to depression and 0.30 to mixed anxiety and depression. Another meta-analysis (Newby et al., 2015) studied the effect of transdiagnostic psychological treatment for depression and anxiety compared to waitlist, TAU and another psychological treatment, showing a large effect size (Hedges’ *g*) for both depression (*g* = 0.91) and anxiety (*g* = 0.85). Regarding UP, an RCT obtained an effect size (Hedges’ *g*) of 0.56 and 1.11 for anxiety and depression, respectively, using this treatment compared to waitlist (Farchione et al., 2012). Additionally, the findings of a recent meta-analysis about the effect of conventional CBT compared to waitlist, TAU or placebo showed an effect size (Hedges’ *g*) of 0.75 for depression, 0.80 for generalized anxiety disorder, 0.81 for panic disorder and 0.88 for social anxiety disorder (Cuijpers et al., 2016). By last, another meta-analysis examined the efficacy of combined therapies (psychotherapy plus pharmacotherapy) in comparison to pharmacotherapy for the treatment of anxiety disorders and depression (Cuijpers et al., 2014). The findings of this study showed an effect size (Hedges’ *g*) of 0.51 in favor of the combined therapy using CBT compared to pharmacotherapy only.

Taking into account these results, a generalized medium effect size of 0.6 (Cohen’s *d*) was assumed to detect differences between the interventions and TAU. Due to software to calculate the sample size for analysis using mixed linear models is not available, we established the sample size for analysis of variance using the *f* index trough G^∗^Power. Therefore, we assumed an effect size of 0.3 (*f* index), equivalent to Cohen’s *d* = 0.6. Thus, with a statistical power of 0.80 and an alpha level of 0.05, a total sample of 140 participants will be required (28 subjects in each group). With the objective of controlling the lack of participants throughout intervention and evaluation process, based on previous studies (Farchione et al., 2012; Ito et al., 2016), we assumed an abandonment rate of 15%. Consequently, we require to include 165 participants in our study (33 participants per group).

### Target Population, Procedure, Randomization, and Blinding

We will include in our study patients with depression, anxiety or somatization disorder of mild to moderate severity. The detection of cases will be done from primary care by a GP. Patients that present these characteristics are encouraged to participate in the trial by de GP. Individuals who consent to participate and write the informed consent will receive the baseline assessment. If all eligibility criteria are fulfilled, the patient will be assigned randomly to one of the five groups according to a computer-generated allocation sequence (ratio: 1.1.1.1.1.). The randomization sequence will be generated prior to the recruitment of participants and will be conducted by a researcher uninvolved in the assessments of the study. At the end of treatment, there will be a post-treatment assessment and at 6-month follow-up. A single-blind process will be applied. The researcher in charge of the process of evaluation of the participants at the post-treatment and follow-up assessments are blinded to the intervention condition.

### Eligibility Criteria

#### Inclusion Criteria

In the study may be included any person between 18 and 65 years of age with a DSM-5 diagnosis of generalized anxiety disorder, agoraphobia, panic disorder, specific phobia, social anxiety (social phobia), anxiety disorder not otherwise specified, dysthymia, mayor depressive disorder, depressive disorder not otherwise specified and/or somatic symptom disorder. Besides, patient must meet predetermined cutoff points of mild to moderate severity level in at least one of the following assessment measures utilized: GAD-7 ≥ 10; PHQ-9 ≥ 10; PHQ-15 ≥ 5; PHQ-PD ≥ 8.

#### Exclusion Criteria

Patients with severe mental disorders (bipolar disorder and/or psychotic disorder), severe depression (PHQ ≥ 20), recent suicide attempts and/or current suicidal ideation, concurrent substance use disorder (alcohol, cannabis, stimulant, hallucinogen, and/or opioid), personality disorders, suspected or obvious mental retardation, and people who are taking pharmacological drugs that interferes with the Central Nervous System will be excluded.

### Ethics Statement

The study protocol (PSI2014-56368-R) has been authorized by the Ethics and Clinical Research Committee of the Ministry of Health of the Andalusia Government (Spain) and has been registered at clinicaltrial.gov with the number NCT03286881. This study is compliant with the General Data Protection Regulation (GDPR) of the European Union, potential study participants must provide written informed consent before they can be included in the study. The participants will receive written information regarding the study, including randomization to one of the study groups and the possibility of ending their participation at any time without disadvantages, before obtaining their informed consent. Participation in the study does not entail any danger for the participants except those related with pharmacological intervention. The engagement is voluntary and confidentiality is guaranteed.

### Interventions

#### Brief Psychological Treatment Based on UP

This intervention protocol consists of eight sessions according to an adaptation to brief format of the UP for the transdiagnostic treatment of EDs (Boisseau et al., 2010; Ellard et al., 2010; Barlow et al., 2011) and the NICE guideline “*Common mental health disorders”* (National Institute for Health, and Clinical Excellence [NICE], 2011). The treatment is developed by clinical psychologists in Specialized Care (SC). Each session has specific objectives:

S1. *Motivation for change and commitment to treatment*. The aim of the first session is to improve motivation for change analyzing the advantages and disadvantages of changing and defining determinants life goals.
S2. *Understanding the function of emotions*. The second session gives information regarding the adaptive functions of emotions, introduces the concept of Emotion Driven Behaviors (EDBs) and distinguishes between thoughts, physical sensations and behaviors related to emotions.
S3. *Emotional awareness training*. The third session aims to introduce and to practice emotional awareness centered on the present, without judging.
S1. *Cognitive appraisal*. The objective of the fourth session is to teach appropriate thinking patterns, showing how to identify maladaptive ways of thinking and how to modify them.
S4. *Emotional avoidance and EDBs*. The purposes of this session are to teach patients that emotional avoidance strategies favor the development and maintenance of EDs and help them to change their own maladaptive behaviors for others more appropriate and functional.
S5. *Tolerance to physical sensations*. This session aims for the patient to get used to the physical sensations by performing exercises that causes these physical sensations, such as breathing through a straw.
S6. *Interoceptive and situational emotional exposure*. The main of this session is to expose the patient to internal and external triggers that produce intense emotional reactions, in order to improve the habituation to emotions and decrease avoidance behavior.
S7. *Conclusion and relapse prevention*. The objectives are to discuss the skills learned throughout the treatment, to identify the skills that should continue to be practiced in the future and to instruct how to face future situations.

#### Conventional Psychological Treatment

Patients in this group will receive the usual psychological treatment established within the healthcare process “Anxiety, Depression, and Somatizations” of the Andalusian Public Health Service (Spain) (Díaz del Peral et al., 2011). This treatment will be conducted in a range between 12 and 24 sessions of traditional CBT applied in a maximum period of 8 months. The treatment will be developed by clinical psychologists in Specialized Care (SC).

#### Conventional Psychological Treatment Plus Pharmacological Treatment

Patients in this group will receive the conventional psychological treatment based on traditional CBT, commented above, plus pharmacological treatment. The psychological treatment will be implemented by clinical psychologists and the pharmacological treatment will be prescribed by psychiatrist both in SC. This treatment will be carried out in a maximum period of 8 months.

#### Minimum Intervention Based on Basic Psychoeducational Information, Counseling, and Bibliotherapy

Patients in this experimental group will receive minimum psychological intervention based on basic psychoeducation, counseling and bibliotherapy by their GPs in PC. Previously, the GPs will have been trained by a clinical psychologist in how to give the patients basic information about their disorders, the use of strategies to face the basic symptoms of EDs without pharmacotherapy, and the use of bibliotherapy as support.

The minimum intervention will be conducted in a range between two to five sessions around 20–30 min long. Each session will be integrated by three components: (a) active listening and counseling; (b) psychoeducation about functioning and regulation of emotions; and (c) guided bibliotherapy. As a material for bibliotherapy, self-help guides of Andalusian Public Health Service^1^ (Spain) will be used considering the main symptoms of each patient.

#### TAU

The participants included in this experimental condition will receive the TAU prescribed by the GP in PC, based on pharmacotherapy exclusively. The GP will prescribe the TAU to the patient in a regular consultation. This consultation will usually consist of a session between 5 and 7 min in which the GP will evaluate the psychological and physical symptoms of the patients and will prescribe psychotropic drugs. This treatment will be carried out in a maximum period of 8 months.

## Proposed Analysis

The data will be analyzed following both intention-to-treat (ITT) and per protocol approaches. Firstly, ANOVA or chi-squared will be performed to compare the demographic variables and outcomes measures at baseline. Second, to examine longitudinal changes over time (baseline, posttreatment and follow-up) and between-group differences on these changes we will use linear mixed models, inasmuch as these models are more accurate than repeated-measures ANOVAs (Gueorguieva and Krystal, 2004). In the ITT analysis (all patients randomized are included in the analysis), the incomplete or missing data will be considered using the maximum likelihood estimation method. Additionally, Cohen’s *d* will be calculated to determine the size of between-group effects. Last, we will estimate the Incremental Cost-Effectiveness Ratio (ICER) to compare the relationship between costs and effectiveness of different interventions.

## Anticipated Results

This research protocol pretends to highlight the importance of adapting treatments to real contexts of application, and their adjustment to brief formats of limited time that maximize the cost-benefit of implemented care, as other authors suggest (Kazdin, 2008; Moriana and Martínez, 2011), besides contributing to the dissemination of psychological treatments in primary and specialized care.

The principal objective of this work is to examine the effectiveness and the efficiency of the brief psychological treatment in a sample of patients with EDs, compared to other four treatments: conventional psychological treatment, conventional psychological treatment plus pharmacological treatment, minimum intervention based on basic psychoeducational information, and pharmacological treatment. Although we think that psychological treatment (brief and conventional) will be more effective than pharmacological treatment, as suggested by previous results with patients with EDs (Heuzenroeder et al., 2004; Watts et al., 2015), we expect that “conventional psychological treatment plus pharmacological treatment” will be the most effective treatment. This findings will be compliant with other studies that indicate that the combined treatment is superior in efficacy to CBT and pharmacotherapy alone in both anxiety disorders (Furukawa et al., 2007; Van Apeldoorn et al., 2008) and depression (Keller et al., 2000; Pampallona et al., 2004).

Due to brief psychological therapies have obtained similar results to conventional psychological therapies (Miller, 2000; Bloom, 2001; Lyons and Low, 2009; Nieuwsma et al., 2012), proving effective in reducing anxious-depressive symptoms (Koutra et al., 2010; Bernhardsdottir et al., 2013; Saravanan et al., 2017), and UP have demonstrated its effectiveness to the treatment of EDs (Barlow et al., 2004; Brown and Barlow, 2009; Farchione et al., 2012), we hope that brief psychological treatment based on UP will be the most efficient treatment and as effective as conventional psychological treatment. However, although there is evidence suggesting that the patient’s improvement, after being treated with brief psychological therapies, remains long after treatment (Hamdan-Mansour et al., 2009; Vázquez et al., 2012), some authors question the maintenance of long-term results (Seekles et al., 2013). Therefore, the results of this study will be added to the evidence of efficacy for brief psychological treatments for EDs right after the treatment and at 6-month follow-up.

By last, we think that “minimum intervention based on basic psychoeducational information, counseling and bibliotherapy” will be more effective than pharmacological treatment for participants included in this study, but will get worst results that different versions of psychological treatment. Due to this kind of interventions are not sufficiently represented in controlled research (Labrador et al., 2000), the results of this work could contribute to the dissemination of minimum psychoeducational interventions to address the treatment of EDs in the PC context.

In last years, new intervention modalities are gaining consensus for the treatment of mental disorders, such as the stepped care approach. This treatment modality consists of apply progressively low and high intensity interventions (Turpin et al., 2008) and is based in two fundamental principles. Firstly, the treatment should produce positive results with the least burden for the patient. Secondly, the results of the patients should be reviewed systematically to decide about the need to move to another level of treatment.

The stepped care model can be implemented in two different ways. Firstly, the purely stepped approach assigns low intensity treatment to all patients and proposes a more intense intervention for patients who do not benefit low intensity treatment. On the other hand, in the stratified approach, patients are initially assigned to different levels of intervention according to an assessment of the symptoms and risks presented (Turpin et al., 2008).

Some recent studies have proved the efficacy of the stepped care model for the treatment of mental disorders. For example, Salomonsson et al. (2018) examined the utility of a purely stepped approach for the treatment of EDs in a primary care setting. These authors showed that the 40% of patients improved with a low intensity treatment. Besides, with this model of treatment less than six sessions were needed per patient, obtaining an overall improve of 63%. Another study (Tasca et al., 2018) tested the usefulness of this approach of intervention for the treatment of binge eating disorder. The findings of this study indicated that the low intensity treatment reduced significantly the symptoms of patients and that a more intensive intervention did not reduce the symptoms more than the low intensity treatment.

In this line, if all the treatments included in our study prove to be effective for addressing EDs, may be used on a stepped care model ordered by the level of intensity. Thus, the minimum intervention based on basic psychoeducational information may be first step of the treatment, followed by brief psychological treatment, conventional psychological treatment and conventional psychological treatment plus pharmacological treatment.

## Limitations

This study presents several limitations. The first one is related to the design of the study, since the interventions included in the protocol are composed by a different number of sessions. Nevertheless, it is necessary to include interventions with different length to determine whether the treatment effect varies depending on the intensity and duration. Besides, except for the “brief psychological treatment based on UP” group, the number of sessions or weeks of interventions within a certain group could vary from one patient to another. However, we will make sure that each patient is evaluated 6 months after the end of the treatment (follow-up assessment).

As a limitation inherent in this type of studies, considerable dropout rate during the intervention process are expected, as well as a substantial missing data to follow-up. Nevertheless, participants will be animated by phone or email to continue with the treatment or to participate in the follow-up assessment. In addition, the anxious-depressive symptoms could be attenuated by the passage of time.

Other limitations may be related to the recruitment difficulties. Due to the participants must be diagnosed with a DSM-5 disorder and must meet predetermined cutoff points, may be recruitment problems related to the difficulty that participants completely met the eligibility criteria. Besides, patients may refuse to be treated by a different intervention than usual.

## Conclusion

This study describes an adaptation to brief format of the UP for the transdiagnostic treatment of EDs. If this RCT proves that brief psychological treatment is as effective as conventional psychological treatment and that both treatments are more effective than pharmacological treatment, the results of this RCT may encourage the implementation of brief therapies in the PC context. This implementation could lead to the decongestion of the public health system, the treatment of a greater number of people with EDs in a shorter time, the reduction of the side effects of pharmacological treatment and a possible economic savings for public purse.

## Author Contributions

JM is the principal investigator of the trial and was primarily responsible for the design and development of the RCT. MG-L, JC, and JV contributed to the study design. JV supervised the study therapists. MG-L, JC, and JM drafted the manuscript. JV contributed to editing the manuscript and read and approved the final version of the manuscript.

## Conflict of Interest Statement

The authors declare that the research was conducted in the absence of any commercial or financial relationships that could be construed as a potential conflict of interest.

## Footnotes

1 The self-help guides of Andalusian Health Service can be consulted and downloaded from the following link: http://www.juntadeandalucia.es/servicioandaluzdesalud/principal/documentosacc.asp?pagina=gr_smental_23_12_gauto
